# Dr. Homer G. Newton

**Published:** 1915-11

**Authors:** 


**Dr. Homer G. Newton,** N. Y. University 1863, died at his home in Sherburne, Chenango County, October 11, aged 79. He was a surgeon in the U. S. army during the Civil War, one of the founders of the Brooklyn Eye and Ear Hospital and, for many years, a practitioner of Brooklyn.

